# Editorial: Biotechnological Production and Conversion of Aromatic Compounds and Natural Products

**DOI:** 10.3389/fbioe.2020.00646

**Published:** 2020-06-19

**Authors:** Nils J. H. Averesch, Oliver Kayser

**Affiliations:** ^1^Department of Civil and Environmental Engineering, Stanford University, Stanford, CA, United States; ^2^Universities Space Research Association, NASA Ames Research Center, Moffett Field, CA, United States; ^3^Department of Technical Biochemistry, Technical University of Dortmund, Dortmund, Germany

**Keywords:** aromatics, shikimate pathway, natural products, metabolic engineering, degradation

Building blocks of aromatic nature are among the most important bulk-feedstocks in the chemical industry, the majority (by volume) serving as precursors for polymeric materials. As these compounds are commonly derived from petrochemistry, obtaining them is increasingly becoming a matter of costs and sustainability. Biochemistry gives rise to a wealth of compounds that can potentially substitute or replace current petroleum-based chemicals or be used for novel applications. This includes bio-replacements for commonly fossil fuel-derived aromatics, as well as naturally produced secondary metabolites. Also, a great number of natural products and secondary metabolites, which are valuable in food- and pharma-industry, are aromatics. Furthermore, for fine chemicals and pharmaceuticals, which need to be produced with high purity or selectivity, cost-competitiveness is much less of a critical factor. This is a particular chance for biotechnology, especially for compounds, which are impossible or infeasible to produce via chemical synthesis.

Aromatics have great potential for bio-based production, as biochemical pathways like the polyketide biosynthesis or the shikimate pathway give rise to a wealth of aromatics and aromatics-derived compounds, with diverse applications in the chemical-, pharma-, cosmetic-, and food-industry. To achieve commercial viability, processes need to be competitive, as determined by the three factors: titer, yield, and rate (Averesch and Krömer, 2018). Therefore, in addition to metabolic engineering, optimization of the process is also imperative, comprised of reactor-design and -operation and has been included as subject in this Research Topic.

Aromatic compounds of interest may be produced microbially outgoing from sugar-based carbon-sources, while many pharmaceutically utilized natural products are still plant-derived. To supply the growing demand of many of these products while ensuring affordability, as well as to uphold a constant supply chain (tolerant to environmental factors such as weather and climate), heterologous production of natural products in microbes is sought after. For sustainability reasons, as well as a potential cost advantage, biotechnology is also increasingly adapting non-edible carbon-sources for production. This can be lignocellulosic biomass, of which the lignin part is especially attractive for production of aromatics, as it can be de-polymerized to directly obtain aromatic compounds. In this context, the degradation and recycling of spend materials, like polyethylene terephthalate, is also of interest, not only to achieve sustainability but also for environmental protection.

This Research Topic was initiated to expand upon a review on biotechnological aromatics production, which ranks metabolic engineering strategies by establishing thresholds of yields, titers and rates for commercial viability (Averesch and Krömer, 2018). Collecting contributions from leaders in the field, we strived to spotlight recent concepts and current trends that shape the future for biotechnological production of aromatic compounds. Deliberately also including research from more peripheral areas, we intended to expand the horizon and show where the field may be headed. Calls for papers resulted in 10 accepted manuscripts, of which eight are original research and two review articles. Topics cover a broad research spectrum, from metabolic engineering for production of compounds valuable in chemical-, food-, and pharmaceutical-industry, including process development and optimization, to studies on mechanisms of degradation, conversion and valorization of aromatic bio-products. Especially the diversity of approaches and deployed organisms, shows that a paradigm shift beyond the established model organisms is taking place.

Two articles review recent advances in microbial production of aromatic compounds: one with a special emphasis on metabolic engineering strategies, as well as bioprocess optimization (Braga and Faria); the other putting special emphasis on melanins (which have applications in the pharmaceutical, cosmetic, optical, and electronic industries) and strategies for their recombinant production (Martínez et al.).

Metabolic Engineering studies utilizing rational strain design are most prominently featured in this Research Topic, three of which are targeting chemical building-blocks for polyesters: through genetic modification of *Pseudomonas taiwanensis*, 4-hydroxybenzoic acid is derived via tyrosine (Lenzen et al.)—an interesting alternative to the more conspicuous pathway where 4-hydroxybenzoic acid is obtained directly from chorismate. In a study more focused on optimization of physiological parameters and process design, conversion of ferulic acid to vanillin is investigated deploying whole-cell biocatalysts with growth arrested *E. coli* (Luziatelli et al.). In another *E. coli* study, deep strain engineering leads to significantly enhanced production of the central shikimate-pathway intermediate 3-dehydroshikimate; in combination with process design this allows downstream conversion into significant amounts of muconic acid (Choi et al.).

Microbial biotechnology has relevance beyond the common carbon-biochemistry, as shown by the fermentative production of natural product 7-bromo-L-tryptophan, a precursor for the proteasome inhibitor and potential anti-cancer agent TMC-95A, which is explored through engineering of *Corynebacterium glutamicum* for the bromination of tryptophan (Veldmann et al.).

Besides bacteria, also fungi have their advantages for production of aromatics: performing Metabolic Engineering on the yeast *Saccharomyces cerevisiae* for production of ergothioneine, an antioxidant, nutraceutical, non-proteinogenic amino acid and not-so-usual aromatic compound (aromatic heterocycle), highlights the diversity of interesting aromatics and the emerging ability to produce these biotechnologically (van der Hoek et al.).

Complementary to the *de-novo* production of aromatics from sugars, two studies evolve around lignin as alternate substrate and biochemically more direct source of aromatic compounds: one study investigates the transcriptional response of the white-rot fungus *Dichomitus squalens* expressing extracellular enzymes during lignin degradation (Kowalczyk et al.). Another study looks at the regulation of degradation of the lignin building-block cinnamic acid in the filamentous fungus *Aspergillus niger* (Lubbers et al.).

Finally, an economic evaluation of a process-model for production of aromatic compounds, sheds light on potential commercial competitiveness and environmental impact, with unexpected and therefore likely often overlooked outcomes (Krömer et al.).

Further reading includes another recent review on “Metabolic engineering of microorganisms for production of aromatic compounds” (Huccetogullari et al., 2019), as well as related Research Topics:

1. Aromatic Amino Acid Metabolism
2. From Biomass-to-Advanced Bio-based Chemicals and Materials.

## Author Contributions

NA conceived of the idea for the Research Topic and served as editor. OK served as co-editors for the Research Topic. NA wrote the editorial, with editing help from OK. Both authors contributed to the article and approved the submitted version.

## Conflict of Interest

The authors declare that the research was conducted in the absence of any commercial or financial relationships that could be construed as a potential conflict of interest.
